# AiiM Lactonase Strongly Reduces Quorum Sensing Controlled Virulence Factors in Clinical Strains of *Pseudomonas aeruginosa* Isolated From Burned Patients

**DOI:** 10.3389/fmicb.2019.02657

**Published:** 2019-11-14

**Authors:** Luis Esaú López-Jácome, Georgina Garza-Ramos, Melissa Hernández-Durán, Rafael Franco-Cendejas, Daniel Loarca, Daniel Romero-Martínez, Phuong Thi Dong Nguyen, Toshinari Maeda, Bertha González-Pedrajo, Miguel Díaz-Guerrero, Jorge Luis Sánchez-Reyes, Dánae Díaz-Ramírez, Rodolfo García-Contreras

**Affiliations:** ^1^Laboratorio de Bacteriología, Departamento de Microbiología y Parasitología, Facultad de Medicina, Universidad Nacional Autónoma de México, Mexico City, Mexico; ^2^Laboratorio de Infectología, Centro Nacional de Investigación y Atención de Quemados, Instituto Nacional de Rehabilitación, Mexico City, Mexico; ^3^Laboratorio de Fisicoquímica e Ingeniería de Proteínas, Departamento de Bioquímica, Universidad Nacional Autónoma de México, Mexico City, Mexico; ^4^Department of Biological Functions Engineering, Gradute School of Life Sciences and System Engineering, Kyushu Institute of Technology, Kitakyushu, Japan; ^5^Departamento de Genética Molecular, Instituto de Fisiología Celular, Universidad Nacional Autónoma de México, Mexico City, Mexico

**Keywords:** AiiM lactonase, virulence factors, *Pseudomonas aeruginosa*, quorum quenching, burned patients, anti-virulence therapy

## Abstract

*Pseudomonas aeruginosa* is an opportunistic bacterium associated with healthcare infections in intensive care units (ICUs), ventilator-associated pneumonia (VAP), surgical site infections, and burns. This bacterium causes 75% of death in burned patients, since it can develop a persistent biofilm associated with infections, express several virulence factors, and antibiotic-resistance mechanisms. Some of these virulence factors are proteases such as elastase and alkaline protease, or toxic metabolites such as pyocyanin and is one of the few microorganisms able to produce cyanide, which inhibits the cytochrome oxidase of host cells. These virulence factors are controlled by quorum sensing (QS). In this work, 30 *P. aeruginosa* clinical strains isolated from burned patients from a tertiary hospital in Mexico City were studied. Antibiotic susceptibility tests were done, and virulence factors (elastase, alkaline protease, HCN, and pyocyanin) were determined in presence of an N-acylhomoserine lactonase, AiiM able to hydrolyze a wide range of acyl homoserine lactones. The treatment reduced significantly the activities of elastase and alkaline protease, and the production of pyocyanin and HCN in all producer strains but not the secretion of toxins through the type III secretion system. Our work suggests that AiiM treatment may be an effective therapy to combat *P. aeruginosa* infection in burn patients.

## Introduction

*Pseudomonas aeruginosa* is an opportunistic bacteria associated with healthcare infections in intensive care units (ICUs), ventilator-associated pneumonia (VAP), central line-associated blood stream infections, surgical site infections (Cohen et al., 2017), burnt wounds (Fournier et al., 2016), and urinary tract infections, otitis media, and keratitis (Chatterjee et al., 2016; Olivares et al., 2016). In the United States, according to the Centers for Disease Control and Prevention, in 2013 it was estimated that every year around 51,000 health-care infections are associated to *P. aeruginosa*, of which 6,700 are multidrug resistant, causing 440 deaths per year (Centers for Disease Control and Prevention, 2018)^1^. Both *P. aeruginosa* and *Acinetobacter baumannii* complex are the most important, resistant and dangerous microorganisms infecting burnt patients (Tredget et al., 1992; Estahbanati et al., 2002; Turner et al., 2014; Centers for Disease Control and Prevention, 2019). Despite medicine advances, these sorts of complications are still a huge problem to solve, and as a consequence, around 75% of burned infected patients die. Burn infections related to *P. aeruginosa* often promote a faster deterioration allowing the spread of bacteria causing death in weeks and even in days (Mcmanus et al., 1985; Turner et al., 2014). *P. aeruginosa* has a wide arsenal of virulence factors that enable it to colonize and cause infections in the host, the relevance of these virulence factors has been demonstrated using *P. aeruginosa* strains with deficiencies in their production, leading to a reduced ability of colonizing and a lower dissemination in the host (Pavlovskis and Wretlind, 1979; Rumbaugh et al., 2009; Jimenez et al., 2012; Castillo-Juarez et al., 2015). Elastase is a metalloprotease that disrupts several proteins such as: collagen, elastin, immunoglobulins (IgA and IgG), complement components, and cytokines like interferon gamma and tumor necrosis factor alpha (Pavlovskis and Wretlind, 1979; Lyczak et al., 2000; Ben Haj Khalifa et al., 2011). Alkaline protease is also a zinc metalloprotease that inhibits phagocytosis, killing through neutrophils, opsonization, the action of the complement cascade by degrading C3b and is as well related to corneal damage (Howe and Iglewski, 1984; Ben Haj Khalifa et al., 2011; Laarman et al., 2012; Lee and Zhang, 2015). *P. aeruginosa* is one of the few microorganisms that can synthesize cyanide through the oxidative decarboxylation of glycine by hydrogen cyanide synthase enzyme, under micro-aerobic conditions (O_2_ < 5%). HCN is a poison that inhibits respiration by inactivating cytochrome oxidase C (Huber et al., 2016). Another important virulence factor is pyocyanin, a blue phenazine, that promotes oxidative stress, which inhibits ciliary movement and delays inflammatory response due to the damage of neutrophils and apoptosis induction (Ben Haj Khalifa et al., 2011; Lee and Zhang, 2015). In burn injuries, pyocyanin plays an important role because it stimulates colonization, damage of surrounding tissue and promotes dissemination. Furthermore, *P. aeruginosa* strains are often multi-drug resistant, limiting treatment options in healthcare settings around the globe, owing this the World Health Organization classified *P. aeruginosa* as the second more threatening bacterium. Moreover, although new antibiotics are available, each time, resistance against those new drugs quickly appears (Fournier et al., 2016; Shortridge et al., 2017; Karampatakis et al., 2018; Shields et al., 2018). In many pathogenic bacteria, virulence factors are controlled by cell to cell communication known as quorum sensing (QS). *P. aeruginosa* has two QS systems mediated by N-acyl homoserine lactones, Las and Rhl, each one is constituted by three elements, a synthase, a signal receptor and an autoinducer signal. The Las system is formed by LasI which is the synthase, the receptor is LasR and the autoinducer is N-3-oxo-dodecanoyl-L-homoserine lactone, meanwhile the Rhl system is formed by the synthase RhlI, RhlR as receptor and the auto-inducer is N-butyryl-homoserine lactone. These two systems are hierarchically organized and each one of them controls several virulence factors. The Las system regulates the Rhl system and virulence factors such as elastase, protease, exotoxin A, alkaline protease and type II secretion system; while the Rhl system enhances the production of rhamnolipids, hydrogen cyanide, and pyocyanin (Van Delden and Iglewski, 1998; Douzi et al., 2011; Lee and Zhang, 2015). Due to the fast increase in bacterial resistance, alternative strategies such as quorum quenching (QQ) have been proposed. QQ consists of blocking or inhibiting cell to cell communication by obstructing the autoinducer synthases, the signal receptors or by degrading the autoinducers via two enzymatic strategies: disrupting the lactone ring through a lactonase or through the cleavage of the acylic tail by acylases (Defoirdt et al., 2013; Defoirdt, 2018). The aim of this work is to evaluate the activity of AiiM, a lactonase enzyme, in *P. aeruginosa* clinical bacterial strains isolated from burnt patients in a third level center of Mexico City, in order to find out if its utilization could eventually be proposed to treat infected burnt patients.

## Materials and Methods

### Clinical Strains

Randomly 200 strains were selected^2^ from the collection belonging to Infectious Diseases Laboratory at Centro Nacional de Investigación y Atención de Quemados at Instituto Nacional de Rehabilitación Luis Guillermo Ibarra, to avoid genomic redundancy pulsed field, gel electrophoresis was performed and only one strain per clonal group was selected for further experiments. Clinical strains were identified with Vitek 2 Compact^®^ (Biomerieux, France) with Gram negative card identification, some biochemical tests included were oxidase, indole production, growth at 42°C, arginine dihydrolase and glucose oxidation/fermentation. The origin of each clinical isolate is shown in Supplementary Table S1.

### Minimal Inhibitory Concentrations

Minimal inhibitory concentrations were determined according to Clinical and Laboratory Standards Institute^®^ (CLSI) M07-A10 (CLSI, 2015) in 96-well plates. Breakpoints interpretation were made according to the M100 Performance Standards for Antimicrobial susceptibility testing 28th edition (CLSI, 2019). Antibiotics included were amikacin (Sigma Aldrich A1774), gentamicin (Sigma Aldrich G3632), aztreonam (Sigma Aldrich PZ0038), ceftazidime (Sigma Aldrich C3809), cefepime (Sigma Aldrich PHR1763), ciprofloxacin (Sigma Aldrich 17850), levofloxacin (Sigma Aldrich 28266), doripenem (Sigma Aldrich 32138), imipenem (Sigma Aldrich I0160), meropenem (Sigma Aldrich M2574), colistin (Sigma Aldrich C4461), and piperacillin/tazobactam (Sigma Aldrich P8396/T2820). *P. aeruginosa* ATCC^®^ 27853 was used as control as according to CLSI (Supplementary Table S1).

### *las/rhl* Genes Detection

#### DNA Extraction

*Pseudomonas aeruginosa* strains were cultured in 5% sheep blood agar during 18 h at 37°C, and then one single colony was taken and lysed in an Eppendorf tube with 500 μL of TE buffer (10 mM Tris–HCl, 1 mM EDTA, pH 7.5) and were set into a heat block at 95°C for 5 min. Tubes were centrifuged, and the supernatant was added into a new tube.

Genes related to Las and Rhl systems were amplified (Table 1) in a final volume of 25 μL of buffer 1X, 3 mM MgCl_2_, 200 μM dNTP′s, 0.2 μM primer forward and reverse, 0.026 U/μL Taq polymerase (Amplitaq Gold^®^ DNA Polymerase, Applied Biosystems N808-0241, United States). The amplification conditions used were: 95°C 10 min, 95°C 30 s, 58°C 45 s and 72°C 50 s during 35 cycles, 72°C 5 min and finally 4°C (Veriti 96 Well thermal cycler, Applied Biosystems, United States). Amplification products were loaded into a 1% agarose gel stained with SYBR^®^ green I (S7567, Life Technologies, United States) and visualized with Gel DOC^TM^ XR + with Image Lab^TM^ software (Bio-Rad, United States). *P. aeruginosa* PAO1 was used as positive control in each one of the systems and Δ *lasR/rhlR* PAO1 as negative control of transcriptional regulators.

**TABLE 1:** Primers used in this study.

**Primer**	**Tm (°C)**	**Size (bp)**
*lasI*-F 5′-CGCGAAGAGTTCGATAAA-3′	59.7	531
*lasI-*R 5′-GGTCTTGGCATTGAGTTC-3′	58.7	
*lasR*-F 5′-ATGGCCTTGGTTGACGGT-3′	65.9	706
*lasR-*R 5′-GACCCAAATTAACGGCCA-3′	63.7	
*rhlI*-F 5′-TTGCTCTCTGAATCGCTG-3′	61	590
*rhlI*-R 5′-GCCATCGACAGCGGTACG-3′	68.3	
*rhlR-*F 5′-ATGAGGAATGACGGAGGC-3′	63.2	675
*rhlR*-R 5′-CGCGTCGAACTTCTTCTG-3′	62.9	

#### AiiM Purification

AiiM construction was provided by Dr. Toshinari Maeda (Nguyen et al., 2019). Briefly 50 mL *Escherichia coli* M15/pQE30 AiiM was grown overnight (ON) in Luria Bertani broth with 100 μg/mL of carbenicillin (Sigma Aldrich C1389) and 50 μg/mL of kanamycin (Sigma Aldrich K1876), afterward, 10 mL of the ON cultures were taken and inoculated into 1 L of terrific broth with carbenicillin and kanamycin as above described, cultures were incubated at 37°C 220 rpm, optical density (OD) at 600 nm was measured until the culture reached an OD of 0.5 and immediately after, it was induced with 500 μM IPTG. The cultures were incubated at 37°C 220 rpm for 6 h and centrifuged at 10,000 rpm for 40 min. Pellets were resuspended in 40 mL of purification buffer (50 mM NaH_2_PO_4_, 300 mM NaCl and 10% glycerol, pH adjusted to 8.0) and 500 μM PMSF (Sigma Aldrich P7626), sonicated (45% amplitude for 45 s and 2 min of rest, all this 10 times; Ultrasonic processor, Cole Parmer) and centrifuged at 10,000 rpm during 40 min. The supernatants were passed through a 0.2 nm filter and loaded onto Protino^®^ Ni-TED resin (Macherey-Nagel, 745200.600) for purification of His-tagged proteins previously equilibrated with 3 column volumes (CV) of 20 mL of purification buffer, then filtered protein extracts were passed through the column, after that, 2 additional CV of purification buffer were passed. Protein was eluted with 150 mM imidazole (Sigma Aldrich I5513) in 2 CV of purification buffer. AiiM fractions with higher purity were selected, concentrated with polyethylene glycol 35 KDa (Sigma Aldrich 946-46) into dialysis tubing cellulose membrane (Sigma Aldrich D9777). Afterward, dialysis was done to remove imidazole using dialysis buffer (50 mM Tris, 300 mM NaCl adjusted at pH 7.5). SDS-PAGE was done to estimate the amount and purity of AiiM. Protein was quantified by its absorbance at 280 nm with NanoDrop 2000 (Thermo Fisher Scientific, United States), using an extinction coefficient (Abs 0.1%) of 1.08. Aliquots of protein were made and stored at −20°C until they were used.

#### AiiM Activity Against N-acyl Homoserine Lactones

To evaluate the HSL lytic activity of the purified AiiM, an analytical assay was developed in an Alliance HPLC system (Waters, United States) with a Symmetry (Waters, United States) C18 Column (75 mm, 3.5 mm). Both short and long acylated chains were included, 1 mM *N-*butyryl-DL-homoserine lactone (C4-HSL; Sigma Aldrich 09945), 1 mM *N*-(3-oxooctanoyl)-L-homoserine lactone (3OC8-HSL;Sigma Aldrich O1764), 1 mM *N*-decanoyl-DL-homoserine lactone (C10-HSL; Sigma Aldrich 17248), 1 mM *N-*(3-oxodecanoyl)-L-homoserine lactone (3OC10-HSL; Sigma Aldrich O9014), and *N-*(3-oxododecanoyl) homoserine lactone (3OC12-HSL; Sigma Aldrich O9139). 60 mM NaOH was used as positive control since it can hydrolyze HSL molecules and the reaction was stopped with the addition of 2N HCl. Several concentrations, from 250, 100, 50, 25, 10, and 5 μg/mL of purified AiiM were tested. Time exposition was also varied; 24 h, 2 h, 1 h, 30 min, 20 min, 10 min, and 5 min. All experiments were performed in triplicate. Chromatographic conditions were: column temperature 25°C, sample temperature 25°C, injection volume 10 μL, flow rate 1 mL/min, detection 205 nm. Elution mixture was made with 50 mM phosphates buffer pH 2.9:acetonitrile. For C4-HSL the relation was 90:10, 3OC8-HSL 60:40, C10-HSL 60:40, 3OC10-HSL 60:40, and 3OC12-HSL 50:50.

#### Bacterial Growth With and Without AiiM

Grow curves were analyzed to test the effect of AiiM on *P. aeruginosa* PAO1 and Δ *lasR/rhlR* PAO1 growth kinetics. A 50 mL flask with 5 mL of LB was inoculated with each one of the strains at an initial OD_600__nm_ of 0.05 with and without 5 μg/mL of AiiM, samples were taken each hour for 12 h.

#### Virulence Factors Determination

Elastase was determined for clinical strains and *P. aeruginosa* PAO1 Δ*lasR/rhlR* and Δ*lasI/rhlI* PAO1 mutants according to methods previously described (Ohman et al., 1980), with some modifications. ON of each clinical strain and control strains were cultured in LB at 37°C 220 rpm and were inoculated at an initial OD_600__nm_ of 0.05 in 5 mL of LB with and without AiiM (5 μg/mL), samples were incubated for 18 h at 37°C and 220 rpm, and centrifuged at 14,000 rpm for 2 min. 50 μL of the supernatant were taken and set into 950 μL of elastase buffer (100 mM Tris–HCl, 1 mM CaCl_2_, pH 7.5) with 2.5 mg of elastin-congo red (Sigma Aldrich reference E0502) as substrate. Tubes were incubated at 37°C, 220 rpm for 2 h, centrifuged at 14,000 rpm for 5 min and the released die in the supernatant was measured at 495 nm with a spectrophotometer SmartSpec Plus (Bio-Rad, United States). All determinations were performed by triplicate.

#### Alkaline Protease

Alkaline protease was determined according to methods previously described (Howe and Iglewski, 1984), with some modifications. ON of each strains were cultured into LB at 37°C 220 rpm and were inoculated in 5 mL of LB at an initial OD_600__nm_ of 0.05 with and without AiiM (5 μg/mL). AiiM protein was added at the beginning of the cultures, samples were incubated for 18 h at 37°C and 220 rpm, and centrifuged at 14,000 rpm for 2 min. 50 μL of supernatant were taken and added into 950 μL of protease buffer with 2.5 mg of Hide-Remazol brilliant blue R (Sigma Aldrich reference H6268) as substrate. Tubes were incubated at 37°C, 220 rpm for 20 min centrifuged at 14,000 rpm 5 min and the supernatant was measured at 595 nm with a spectrophotometer SmartSpec Plus (Bio-Rad, United States).

#### Pyocyanin

For pyocyanin production ON of each clinical and control strains were cultured in LB at 37°C at 220 rpm and inoculated into 5 mL of LB at an initial OD_600__nm_ 0.05 with and without AiiM (5 μg/mL). AiiM protein was added at the beginning, samples were incubated 18 h at 37°C and 220 rpm. One milliliter of supernatant was taken, centrifuged at 14,000 rpm for 5 min, and then 800 μL of supernatant were set into new 1.5 mL conic tube and 400 μL of chloroform was added, each tube was mixed in vortex for 2 min. Tubes were centrifuged for 5 min at 14,000 rpm, 300 μL of the organic phase were taken and deposited into a new tube, 800 μL of 0.2 N HCl were added and mixed for 2 min in vortex then samples were read at 520 nm (Maeda et al., 2012). *P. aeruginosa* PAO1,Δ*lasR/rhlR* PAO1 and Δ*lasI/rhlI* PAO1 were used as positive and negative controls, respectively.

#### HCN Determination

For HCN determination, bacteria were cultured in 3 mL of LB medium in flasks with rubber stoppers at 37°C and 200 rpm for 18 h, after the incubation two needles were inserted in the rubber stopper, one of them was used for pumping air for 1 h and the other to collect the outflow in 5 mL of 4 M NaOH. HCN concentrations were determined as described by Gallagher and Manoil (2001), briefly, samples were mixed with a 1:1 fresh mixture of 0.1 M *o*-dinitrobenzene and 0.2 M *p*-nitrobenzaldehyde both dissolved in 2-methoxyethanol, and following 20 min of incubation at room temperature, the absorbance at 578 nm was determined and compared with a calibration curve made with KCN standards.

#### AiiM Dose Response Curve and Suppression of Its Activity by Exogenous Addition of 3OC12-HSL

For these control experiments, the PAO1 reference strain and the clinical isolate P809 were used. Three independent cultures per strain were inoculated in LB medium at an initial OD_600__nm_ of 0.05 without and with AiiM at 0.5, 1, 2.5, and 5 μg/mL, and incubated 37°C at 220 rpm, supernatants were then obtained and used for the determination of pyocyanin concentration and elastase activity (as described before). In addition another 3 cultures per strain with AiiM 0.5 μg/mL were grown to an OD_600__nm_ of ∼ 1.0, supplemented with a final concentration of 30 μM of 3OC12-HSL, and incubated until 18 h of incubation were completed, supernatants were collected and pyocyanin concentration and elastase activity determined.

#### Long Chain HSL Autoinducer Detection and Its Inactivation by AiiM

To identify autoinducer production and its inactivation by AiiM, each one of the 30 clinical strains were grew up onto MacConkey agar plates, then one colony was taken and inoculated into 5 mL of LB for ON growth. After that, new cultures were inoculated at an initial OD_600__nm_ 0.05 in 5 mL of LB and grown at 37°C, with 220 rpm shaking during 18 h, with AiiM 5 μg/mL and without AiiM enzyme. LB cultures then were centrifuged 14,000 rpm for 5 min. Supernatants were separated in new tubes. For long chain HSL detection *Agrobacterium tumefaciens* NT1 pZLR4 (Shaw et al., 1997) was used as a biosensor strain. Previously the biosensor strain was grown in one liter of M9 medium and incubated at 37°C and 220 rpm for 18 h. Bacteria were concentrated by centrifugation at 12,000 rpm during 5 min to a final volume of 15 mL, then aliquots of 1 mL of concentrated bacteria were separated. M9 agar plates were prepared and before solidification 1 mL of the concentrated biosensor plus Xgal at a final concentration of 40 μg/mL (5-bromo-4-chloro-3-indolyl- ß-D-galactopyranoside, USB corporation, Cleveland, OH, United States) were added for 100 mL of M9 agar. 15 μL of each supernatant (with and without AiiM) were then added onto 6 mm filter paper sterile disk on the M9 agar. All experiments were done by triplicate. 1 mM *N*-decanoyl-DL-homoserine lactone (C10-HSL; Sigma Aldrich 17248), 1 mM *N-*(3-oxodecanoyl)-L-homoserine lactone (3OC10-HSL; Sigma Aldrich O9014) and *N-*(3-oxododecanoyl) homoserine lactone (3OC12-HSL; Sigma Aldrich O9139) were used as positive controls and the molecules treated with AiiM 5 μg/mL as negative controls. Plates were incubated at 28°C and results were observed, a positive reaction associated to the production of long chain HSL was observed as a green halo and inactivation of the signals when the halo was absent.

#### Type III Protein Secretion Profiles

For type III secretion assays *P. aeruginosa* strains (PAO1, PA14, and the clinical isolates H015 and P729) were grown overnight in LB medium. Bacteria were diluted 1:200 into 4 mL of a modified LB medium supplemented with 10 mM MgCl_2_, 0.5 mM CaCl_2_ and 5 mM EGTA (pH 7.4) in the presence or absence of 5 μg/mL of AiiM, and grown at 37°C to an OD_600__nm_ of 0.8 to 1.0. 1 mL of each culture was collected into a microcentrifuge tube and bacteria were pelleted by centrifugation. The resulting pellet was resuspended in 200 μl of 1× Laemmli SDS sample buffer normalized for OD_600__nm_. The supernatant was centrifuged once again and the resulting supernatant was transferred into a clean tube. Supernatant proteins were precipitated overnight at 4°C by adding trichloroacetic acid to a final concentration of 10%, pelleted by centrifugation and resuspended in 20 μL of 1x Laemmli SDS sample buffer containing 10% saturated Tris base normalized for OD_600__nm_. Samples were separated by 15% SDS-PAGE, transferred onto a nitrocellulose membrane and probed for the presence of the effectors ExoS and ExoU by immunoblotting. Detection was performed using the Immobilon Western chemiluminescent HRP substrate (Millipore), and bands were visualized on X-ray films (Carestream MXB Film).

#### ß-Lactams Inactivation

As ß-lactams have a lactone ring, we performed the inactivation disk method to determine whether AiiM would be able to inactivate this kind of antibiotics. Briefly, 2 mL of 0.8% isotonic saline solution (ISS) was added into sterile 12 mm × 75 mm tube, disks of 30 μg ceftazidime (Becton Dickinson, United States), 30 μg cefepime (Becton Dickinson, United States), 10 μg imipenem (Becton Dickinson, United States), and 10 μg meropenem (Becton Dickinson, United States). One set of disks containing ISS was used as negative control, another set of all antibiotics above mentioned with 5 μg/mL of AiiM, and finally, since NaOH can break the lactone ring another set of all antibiotics was used as positive control with 60 mM NaOH. *Escherichia coli* ATCC^®^ 25922 was used as a pansusceptible strain. Disks were incubated for 1 and 10 min, 2 and 24 h. A suspension of 0.5 McFarland was made with *E. coli* ATCC^®^ 25922 and was plated onto Müller Hinton agar (Becton Dickinson, United States), tubes were incubated at 37°C until their use. All experiments were made by triplicate; the inhibition diameter was measured using a Vernier device.

## Results

All clinical isolates were obtained from burned patients infected with *P. aeruginosa*. The most common burn etiology was fire (66.7%) followed by scalds (23.3%) and electrical burns (10%). Medians of hospital length of stay were 53 days (8-303), the mean total body surface area was 40% (10-85%) with a mortality rate of 26.6% (*n* = 8). *P. aeruginosa* strains were isolated from urine (*n* = 8), quantitative biopsies (*n* = 8), blood (*n* = 6), endotracheal aspirates (*n* = 3), bronchoalveolar lavage fluid (*n* = 2), catheter tips (*n* = 2), and qualitative biopsy (*n* = 1).

### *Pseudomonas aeruginosa* Antibiotic Susceptibility Patterns

**FIGURE 1:**
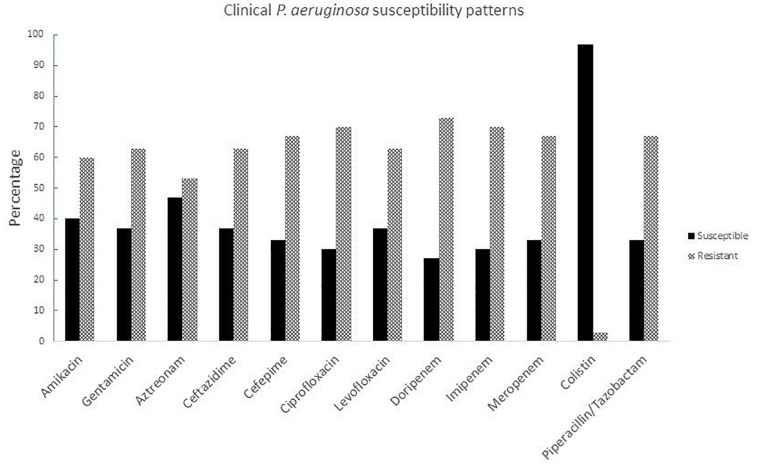
Susceptibility patterns of clinical *P. aeruginosa* strains isolated from burned patients.

Susceptibility tests were carried out for all clinical isolates with different antibiotic families including aminoglycosides, monobactams, cephalosporins, fluoroquinolones, carbapenems, lipopeptides, and ß-lactam combination agents. The strains were resistant to almost all antibiotics except colistin (Figure 1), resistance rates against all antibiotics families were over 50%. The highest resistance rates were for carbapenems which, until recent decades, were the most potent antibiotics against *P. aeruginosa* and other non-fermentative Gram negative rods; therefore colistin represents the last treatment option for these types of infections (Supplementary Table S1).

### Gene Amplification

In order to determine if Las/Rhl systems were present in *P. aeruginosa* isolated from burned patients, PCR was performed using the primers described in Table 1. Results showed that the genes encoding the Las system (*lasI* and *lasR*) and Rhl system (*rhlI* and *rhlR*) were found in all the clinical strains. *P. aeruginosa* PAO1 was used as positive control and Δ*lasR/rhlR* PAO1 was used as negative control (data not shown).

### AiiM Purification

AiiM protein was obtained at a purity of >90% as judged by SDS-PAGE. After elution with 150 mM imidazole, fractions were collected, and those in which AiiM was present were concentrated into dialysis tubing cellulose membrane with polyethylene glycol, then protein concentration was determined and kept at −20°C until used. The purified protein consists of a single band of ≈ 27 kDa, compared with a theoretical molecular mass of 27.2 kDa (Supplementary Figure S1).

### AiiM Activity Against Homoserine Lactones

**FIGURE 2:**
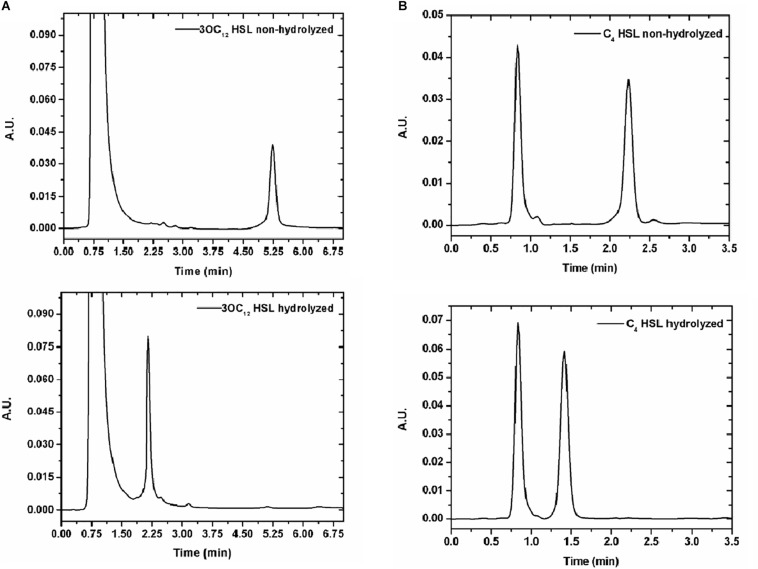
**(A)** HPLC of HSL without (above) and with 5 μg/mL of AiiM (below) after 5 min of exposition. 2A *N-*(3-oxododecanoyl) homoserine lactone. Retention time for non-hydrolyzed HSL is 5.3 min whereas for hydrolyzed is 2.24 min. **(B)** 2B *N-*butyryl-DL-homoserine lactone. Retention time for non-hydrolyzed is 2.29 min whereas for hydrolyzed is 1.42 min.

In order to prove AiiM activity against acylated chains of diverse HSL, cleavage was determined by HPLC. Five HSL autoinducers with both short and long chains were tested (C4-HSL, 3OC8-HSL, C10-HSL, 3OC10-HSL, and 3OC12-HSL). First HSL alone was run to identify retention times (Supplementary Table S2), later the same HSL were treated with 60 mM NaOH to disrupt the lactone ring and finally HSL molecules were treated with AiiM and HPLC experiments were performed under the same conditions. Several AiiM concentrations (250 μg/mL, 100 μg/mL, 50 μg/mL, 25 μg/mL, 10 μg/mL, and 5 μg/mL) were used, in order to identify the lowest one suitable for inhibiting the expression of virulence factors. Each HSL was used at 1 mM. The lowest concentration of AiiM tested (5 μg/mL) was enough to cleave all the HSLs in 5 min (Figure 2 and Supplementary Figure S2) and therefore, this concentration was used for all subsequent experiments.

### AiiM Does Not Affect *Pseudomonas aeruginosa* Growth

Growth curves of *P. aeruginosa* with and without AiiM were done to determine if its addition had any effect in the growth rates. As expected, there was no difference in the growing dynamics between these cultures (Supplementary Figure S3). PAO1 and Δ *lasR/rhlR* strains were used as controls.

### QS-Controlled Virulence Factors Inhibition

**FIGURE 3:**
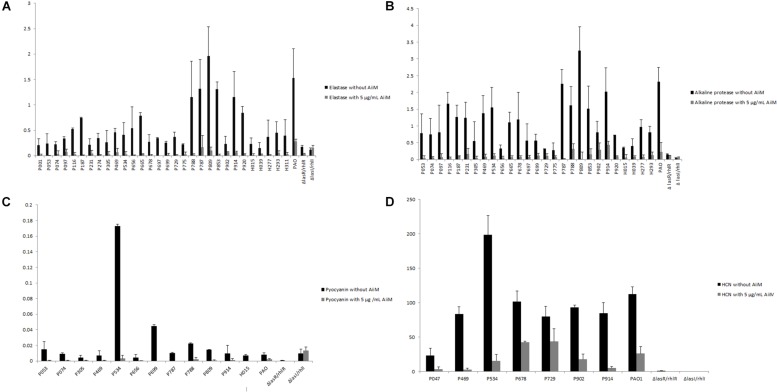
**(A)** Elastase activity, black bars correspond to clinical strains determination without addition of AiiM, gray bars correspond to clinical strains with 5 μg/mL of AiiM with *p* = 0.000002. **(B)** Alkaline protease activity, black bars correspond to clinical strains without AiiM addition while gray bars correspond to strains with the addition of 5 μg/mL of AiiM with *p* = 0.000004. **(C)** Pyocyanin production, black bars correspond to clinical strains without AiiM addition, whereas gray bars correspond to clinical strains with AiiM 5 μg/mL with *p* = 0.001. **(D)** HCN production, black bars correspond to clinical strains without AiiM addition while gray bars correspond to clinical strains with AiiM 5 μg/mL having a *p* = 0.008.

Once it was confirmed that AiiM did not affect *P. aeruginosa* growth, its effect over the expression of the QS-controlled virulence factors was determined. Experiments were classified in two groups, one without AiiM addition, and the other with 5 μg/mL addition of AiiM. For elastolytic activity (Figure 3A), activity was found in 29 clinical samples, while for alkaline protease activity (Figure 3B) there were 27 producing strains; only 12 strains produced pyocyanin (Figure 3C), and seven strains were HCN producers (Figure 3D). At the same time, experiments with AiiM addition were carried out and the same virulence factors were measured. A significant decrease in the production of each virulence factor was found (elastase *p* = 0.000002, protease *p* = 0.000004, pyocyanin *p* = 0.001 and *p* = 0.008 for HCN).

In addition, AiiM dose response experiments using it at 0.5, 1, 2.5 and 5 μg/mL were performed with the reference strain PAO1 and the clinical isolate P809 measuring pyocyanin production and elastase activity, as expected the degree of inhibition of both phenotypes was dependent in the concentration of AiiM for both virulence factors (Supplementary Figure S4). Moreover the effect of 0.5 μg/mL was partially reversed by the addition of 30 μM of 3OC12-HSL (Supplementary Figure S4). In order to verify that the clinical strains had active QS systems and that the inhibitory effect in the expression of QS-dependent virulence factors exerted by AiiM was mediated by the degradation of QS signals. Identification of long chain HSL for each strain was done using the biosensor strain *Agrobacterium tumefaciens* NT1 pZLR4 (Shaw et al., 1997), as expected all strains were long chain HSL producers, moreover also for all strains AiiM at 5 μg/mL was enough to degrade the long chain HSL of all strains as determined with the biosensor strain (Supplementary Figure S5).

### AiiM Does Not Inhibit the Type III Secretion System

Despite its strong inhibitory activity against QS-controlled virulence factors, AiiM had no effect on the secretion of T3SS effectors in both PA14 and PAO1 type strains as well as in the clinical strains P729 and H015 at 5 μg/mL (Supplementary Figure S6) and even using 20 μg/mL (data not shown). These two clinical strains were selected as representative examples of a secretion profile similar to strains PAO1 or PA14, respectively.

### AiiM Does Not Inactivate ß-Lactam Antibiotics

Wang et al. (2010) defined AiiM as a member of the superfamily of alpha/beta hydrolases, this may represent a problem if it has the ability to inactivate the broad spectrum of ß-lactam antibiotics as other carbapenem enzymes do, such as NDM, IMP or VIM. In order to test this, we exposed anti *Pseudomonas* ß-lactam antibiotics to 5 μg/mL of AiiM (Supplementary Figures S7, S8). Nevertheless, AiiM did not degrade any anti *Pseudomonas* ß-lactam antibiotics.

## Discussion

*Pseudomonas aeruginosa* is one of the main bacteria that causes hospital acquired infections in immunocompromised patients and vulnerable ones (Azam and Khan, 2018). *P. aeruginosa* is one of the 12 priority multi-drug resistant bacteria according to the WHO list published in 2017 (WHO, 2017) and belongs to the ESKAPE group, together with *Enterococcus faecium, Staphylococcus aureus, Klebsiella pneumoniae, A. baumannii*, and *Enterobacter* species (Chen et al., 2018). In addition to acquired resistance mechanisms such as carbapenemases (Carmeli et al., 2016), *P. aeruginosa* has many intrinsic antibiotic tolerance mechanisms, for instance low permeability in its external membrane, and expression of several efflux pumps (Malhotra et al., 2018; Ferrer-Espada et al., 2019).

Burn injuries are one of the most common and devastating forms of trauma and patients with serious thermal injury require immediate specialized care in order to minimize morbidity and mortality (Church et al., 2006). *P. aeruginosa* is one of the most frequent bacteria associated to infection in burn patients together with *A. baumannii* (Li et al., 2018). In a recent Mexican study (Garza-Gonzalez et al., 2019), *P. aeruginosa* had around 27% of resistance to carbapenems, in a global context it was one of the main bacteria in 47 Mexican health centers in 20 states, 175/1995 strains were multi-drug-resistant, 165/1995 were possible extreme drug resistant and 87/1995 possible pandrug resistant. In our 30 isolates we had more than 60% of resistance to cephalosporins (ceftazidime and cefepime), carbapenems (dorypenem, imipenem, and meropenem), aminoglycosides (amikacin and gentamicin), fluoroquinolones (ciprofloxacin and levofloxacin), and piperacillin/tazobactam. Moreover, one strain was resistant to colistin, which is the last antibiotic resource.

AiiM showed a wide activity and was able to cut all HSL molecules tested, consistent with a previous report by Wang et al. (2010). Even though we did not analyze it in a quantitative form, we infer a strong activity of AiiM due to its ability to degrade all HSL tested within 5 min of exposition, moreover in our study 5 μg/mL were enough to break down these molecules. One of the main characteristics that a quorum quencher must fulfill is that it should not inhibit bacterial growth (Defoirdt et al., 2013; Defoirdt, 2018) and as expected, AiiM treatment did not affect *P. aeruginosa* growth kinetic.

In contrast, AiiM significantly reduced the four QS-dependent virulence factors tested in our study following a dose response pattern; moreover, others have demonstrated that AiiM had very good activity in a mouse model of acute pneumonia (Migiyama et al., 2013) and reduces methane production in waste sewage sludge (Nguyen et al., 2018). Although to date, the majority of studies with QQ enzymes have been performed only in type strains like PAO1, PA14 (Fetzner, 2015), recently, Guendouze et al. (2017) did the first investigation with *P. aeruginosa* clinical strains isolated from diabetic foot using the lactonase *Sso*Pox with a substitution in the amino acid 263 changing a tryptophan to isoleucine, in order to increase the enzymatic activity, using 0.5 mg/mL of protein, they found some strains with certain tolerance to the *Sso*Pox addition. In our study, AiiM reduced elastase and alkaline protease activities, pyocyanin and HCN concentrations, and we did not find any strain with tolerance against AiiM treatment, in spite that AiiM was used at a 100 times lower concentration than SsoPox. Moreover, AiiM effectivity is much higher than the effectivity of small molecule QS inhibitors such as brominated furanones and 5-fluorouracil, that cannot inhibit QS-virulence factor production of several of the clinical strains tested, and that are very toxic to some of them (García-Contreras et al., 2013, 2015; García-Contreras, 2016; García-Contreras et al., 2016; Guendouze et al., 2017).

Nevertheless, for type III secretion, no inhibition by AiiM was found, which is consistent with recent findings showing that in a Δ*lasR/rhlR* mutant of *P. aeruginosa* PAO1, T3SS effector toxins are secreted at the same levels than in the wild-type strain, demonstrating that this virulence factor is not positively regulated by QS (Soto-Aceves et al., 2019), instead it may be used at low cell densities to establish infections in the host (Hauser, 2009). These highlights the importance of targeting both QS and T3SS to develop robust anti-virulence therapies (García-Contreras, 2016). Moreover, other results indicate that the inhibition of QS systems and T3SS by molecules such as coumarin (Zhang et al., 2018) must be due to independent effects over the QS systems and T3SS.

One possible limitation of the utilization of AiiM and other QQ enzymes for treating *P. aeruginosa* infections is the fact that *lasR* defective mutants are often found in infections and although in principle these mutants will produce low levels of QS-dependent virulence factors, this is not always the case due to a rewiring of the virulence factor regulation (Morales et al., 2017). And these strains could be tolerant against the effect of QQ enzymes.

Since AiiM is a member of the alpha/beta hydrolases superfamily and several antibiotics are inactivated by metallo ß-lactamases, reducing clinical options to treat infections (Hong et al., 2015), we tested if AiiM could cleave these ß-lactamase antibiotics, however, AiiM did not inactivate those tested, and hence it could be safely used in combination with them.

Although *in vivo* tests in burn infection models are lacking, our work suggests that AiiM treatment may be an effective addition for the treatment of *P. aeruginosa* infections, and since research by other groups had shown also the utility of the lactonase *Sso*Pox against clinical isolates from diabetic foot patients *in vitro* (Guendouze et al., 2017) and *in vivo* using an amoeba model (Mion et al., 2019), and in rat pneumonia against the PAO1 strain (Hraiech et al., 2014) lactonase utilization became an strong candidate for its eventual application in the clinical practice, moreover although *in vivo* studies using acylases are scarce, recently it was shown the PvdQ in addition to their inhibitory properties *in vitro* (Sio et al., 2006) and in *Caenorhabditis elegans* model was also able to increase survival, reduce damage and decrease bacterial loads in a pulmonary infection mice model (Papaioannou et al., 2009; Utari et al., 2018). Hence QQ enzymes may be beneficial for the treatment of burn and lung infections as well.

## Conclusion

AiiM showed a strong activity against C4-HSL, 3OC8-HSL, C10-HSL, 3OC10-HSL, and 3OC12-HSL. It reduced elastase and alkaline protease activities as well as pyocyanin and HCN concentrations in all tested clinical strains of *P. aeruginosa* isolated from burned patients and no AiiM tolerant strain was found. However, it had no inhibitory effect against the T3SS.

## Data Availability Statement

All datasets generated for this study are included in the article/Supplementary Material.

## Ethics Statement

*Pseudomonas aeruginosa* clinical strains used in this study were isolated as part of routine clinical hospital procedures to diagnose infection and hence ethical approval was not required, according to the National Institute of Rehabilitation ethical committee. All bacterial isolates were stored as part of laboratory and epidemiology necessities.

## Author Contributions

LL-J, GG-R, MH-D, DR-M, PN, MD-G, DL, JS-R, and DD-R performed the experiments. RF-C, TM, BG-P, GG-R, LL-J, and RG-C designed the study, supervised the project and discussed the results. LL-J wrote the manuscript with input from all authors.

## Conflict of Interest

The authors declare that the research was conducted in the absence of any commercial or financial relationships that could be construed as a potential conflict of interest.

## Footnotes

1 https://www.cdc.gov/hai/organisms/pseudomonas.html

2 http://www.randomization.com
