# Supplementary material for: AiiM Lactonase Strongly Reduces Quorum Sensing Controlled Virulence Factors in Clinical Strains of Pseudomonas aeruginosa Isolated From Burned Patients
Source: Front Microbiol. 2019 Nov 14;10:2657. doi: 10.3389/fmicb.2019.02657 (PMC6868103; doi:10.3389/fmicb.2019.02657)
Supplement: Supplementary file 1 [file Data_Sheet_1.docx]

**Supplementary figures**


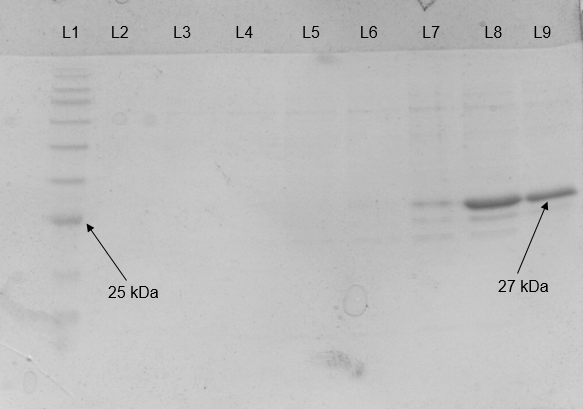


Figure S1. SDS-PAGE of AiiM purification. L1; protein weight marker, L2-L9 purification fractions

**A**


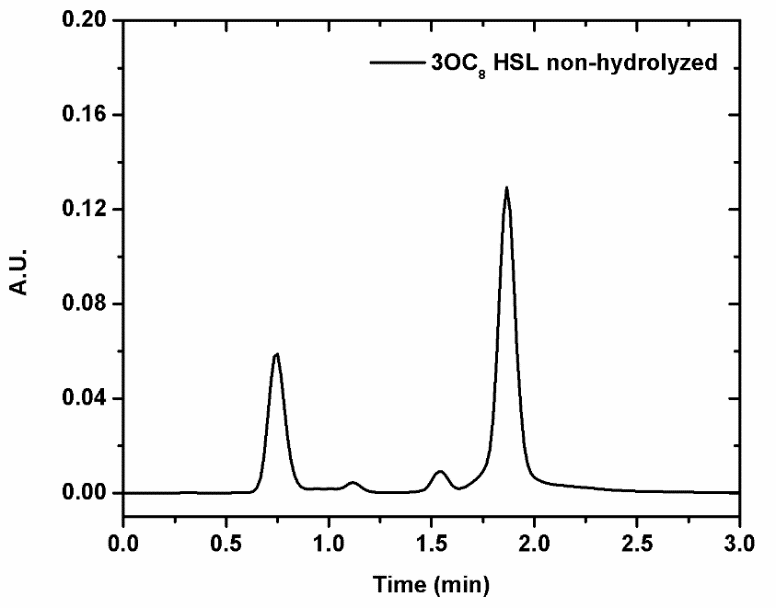

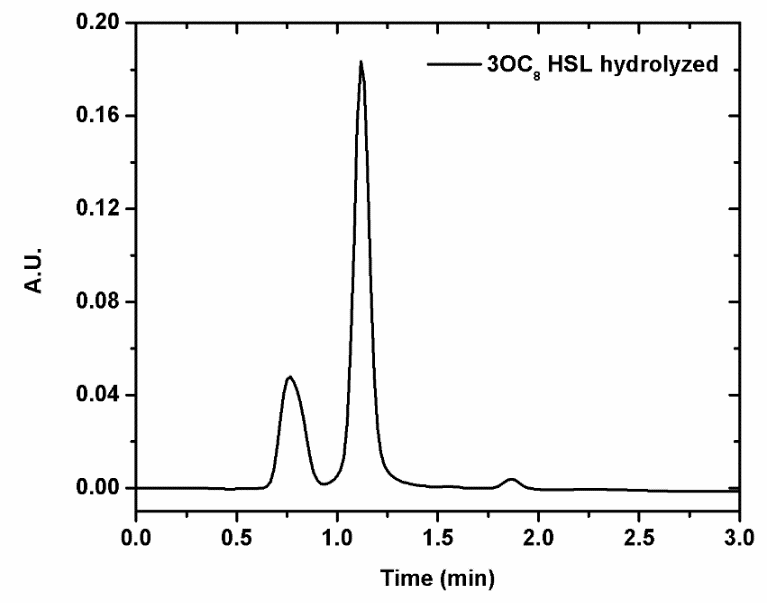

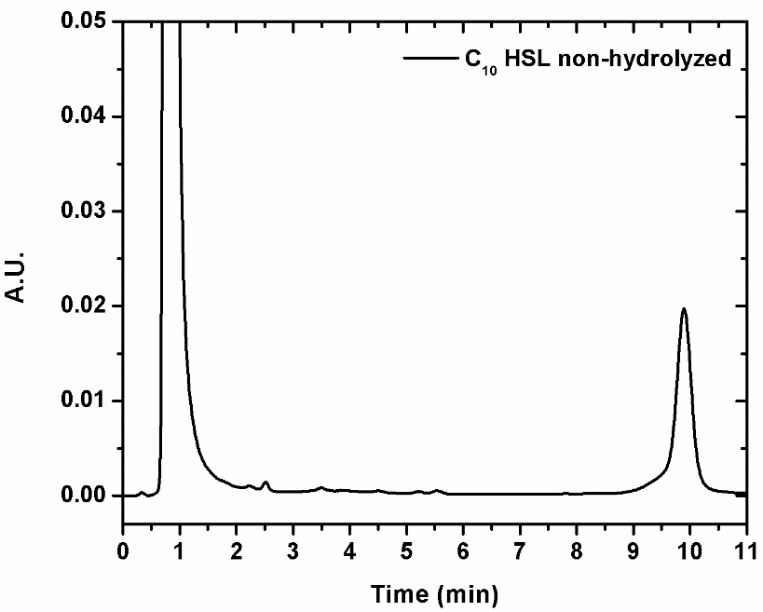

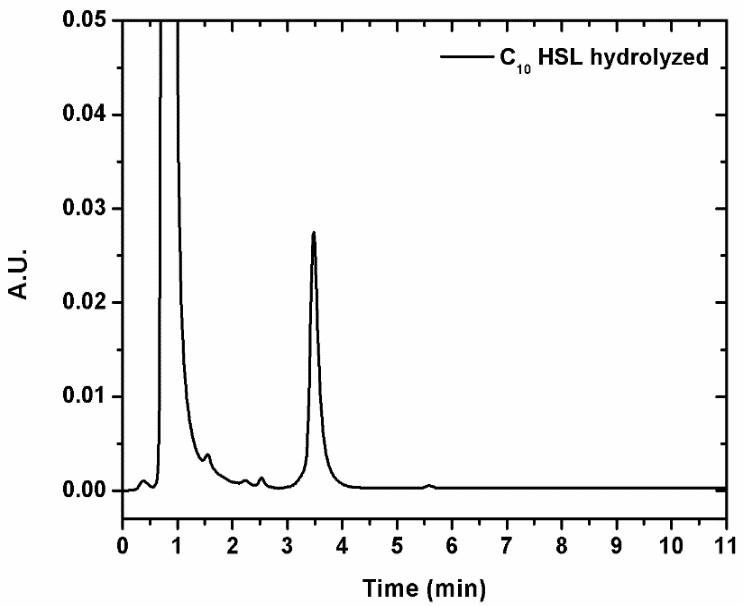

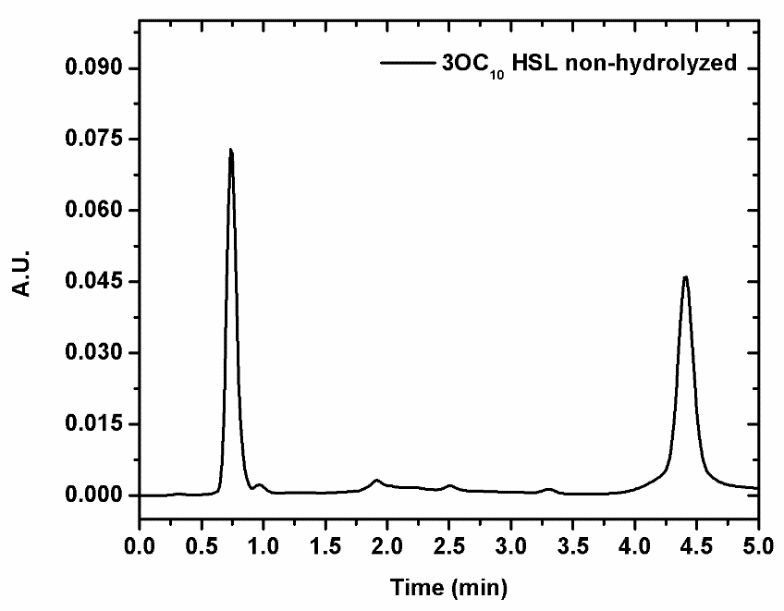

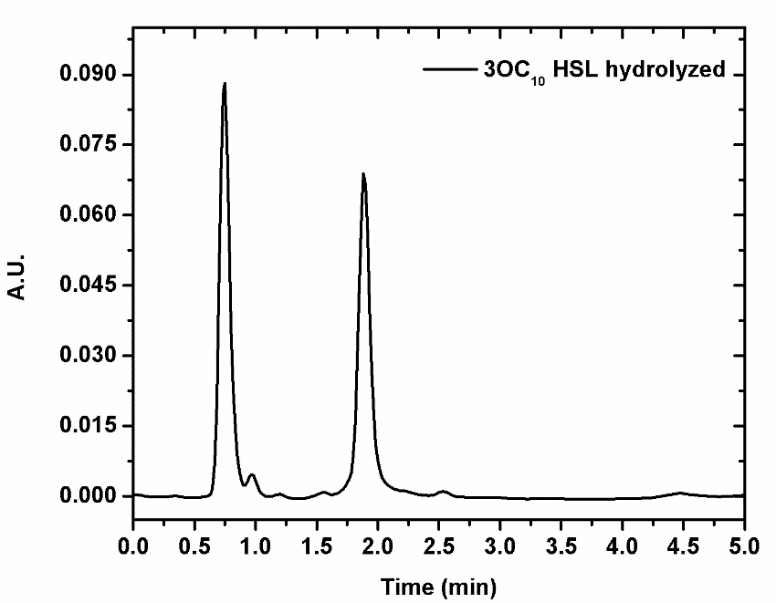


**B**

**C**

Figure S2. HSL Cromatograms without (left) and with 5 µg/mL AiiM (right). A: *N*-(3-oxooctanoyl)-L-homoserine lactone. B: *N*-decanoyl-DL-homoserine lactone and C: *N-*(3-oxodecanoyl)-L-homoserine lactone

Figure S3. *Pseudomonas aeruginosa* kinetic growth. Circles correspond to *P. aeruginosa* PAO1 without enzyme, diamonds correspond to *P. aeruginosa* plus 5 µg/mL AiiM, triangles correspond to Δ*lasR/rhlR* background in PAO1 and finally squares correspond to Δ*lasR/rhlR* background in PAO1 plus 5 µg/mL AiiM.


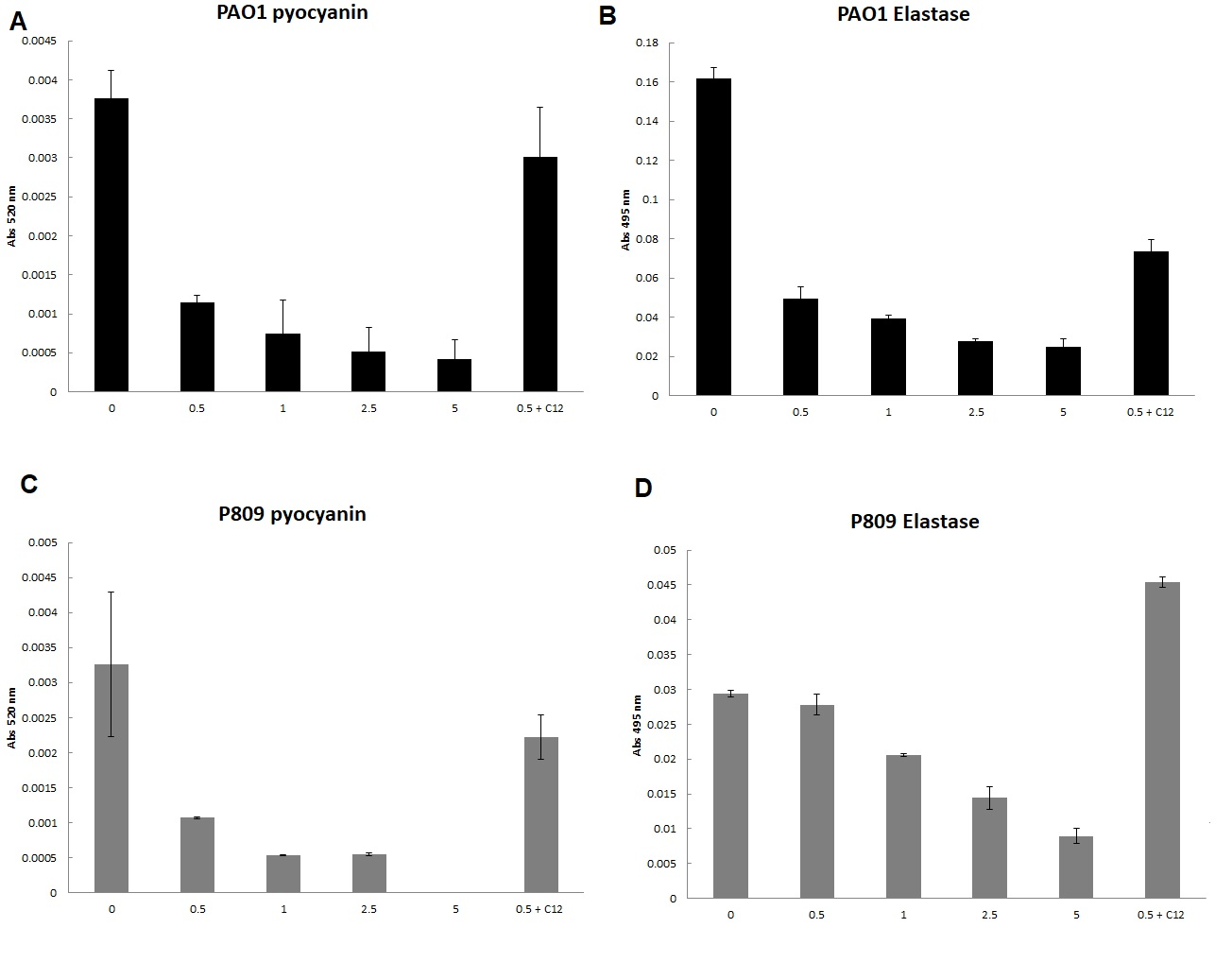


Figure S4.

Effect of increasing concentrations (0. 0.5, 1, 2.5 and 5 µg/mL) of AiiM in A, B) pyocyanin production and C,D) elastase activity of the PAO1 and P809 strains. Experiments were performed in triplicate and the mean standard deviation is shown. The bar labeled as 0.5 + C12 correspond to the treatment with 0.5 µg/mL of AiiM in cultures supplemented with 30 mM of exogenous 3OC12-HSL.


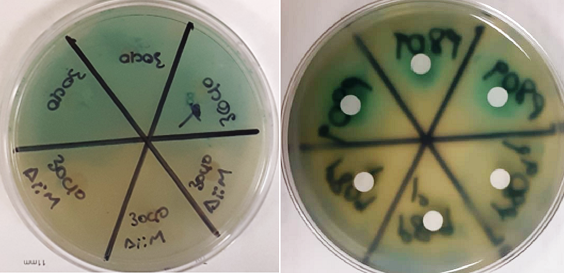


Figure S5. Autoinducer detection and inactivation. Left) above is *N-*(3-oxodecanoyl)-L-homoserine lactone (3OC10-HSL; Sigma Aldrich O9014), below the same HSL molecule but with AiiM, no green pigment green was observed due the degradation of long chain HSL by AiiM. Rigth) 1, 2 and 3 are the strain P089, without AiiM, the presence of a green halo demonstrates that long chain HSL molecules are present, in contrast below the degradative action of AiiM at 5 µg/mL AiiM was demonstrated by the absence of the green coloration. Similar results were obtained for the other 29 clinical strains.


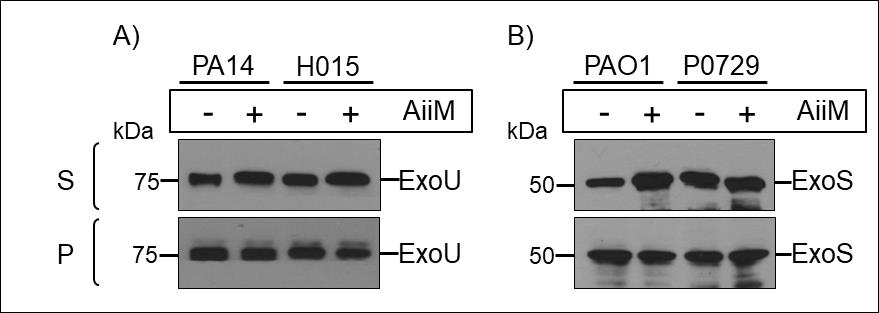


Figure S6. AiiM does not inhibit the secretion or production of T3SS effectors ExoU and ExoS from *P. aeruginosa* type strains PAO1 and PA14, and clinical strains H015 and P0729. The secretion assay was carried out in the presence (**+**) or absence (**-**) of 5 μg/ml of AiiM. A and B) Secreted (S) and total proteins in whole-cell lysates (P) were examined by immunoblotting using anti-ExoU or anti-ExoS polyclonal antibodies. The results shown are representative of three independent experiments.

Figure S7. ß-lactams are not inactivated by AiiM treatment.


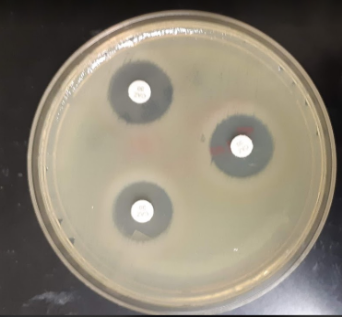

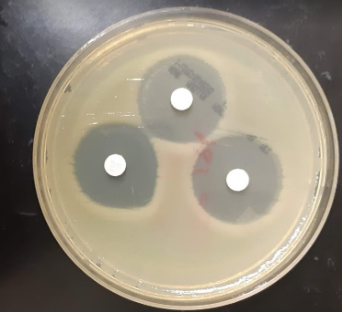

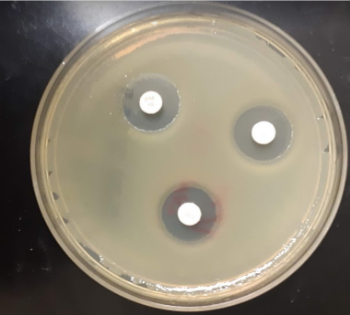

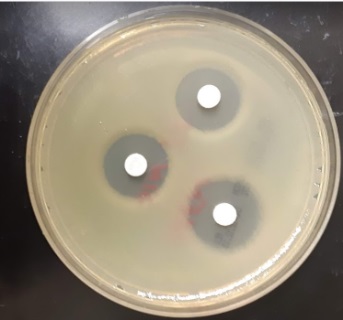


A B C D


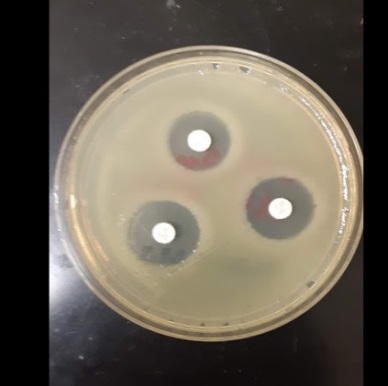

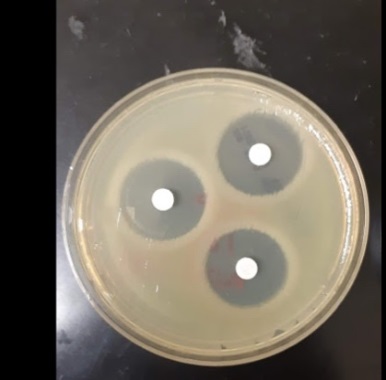

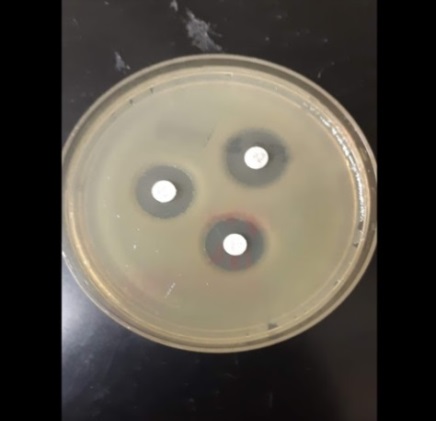

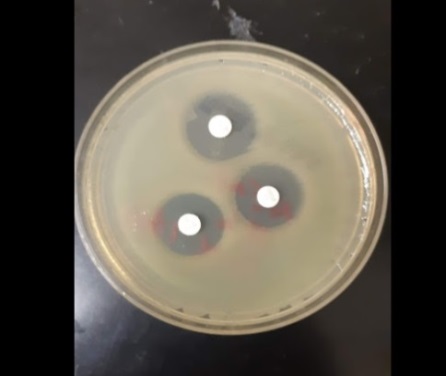


E F G H


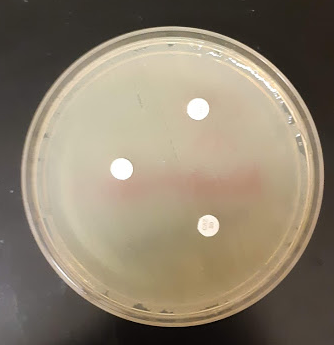

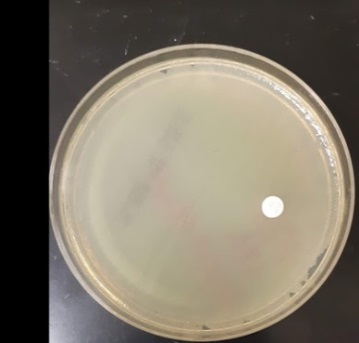

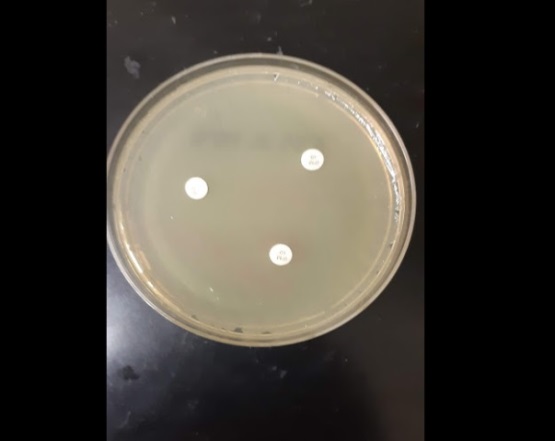

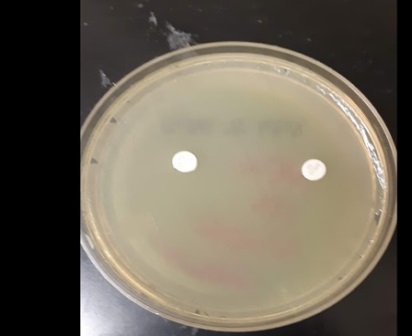


I J K L

Figure S8. ß-lactams inactivation method. A-D ß-lactams without treatment after 24 h of incubation (isotonic saline solution only). A) Ceftazidime, B) Cefepime, C) Imipenem and D) Meropenem. E-H ß-lactams with 5 µg/mL AiiM after 24 h of incubation. E) Ceftazidime, F) Cefepime, G) Imipenem and H) Meropenem. I-L ß-lactams with 60 mM NaOH after 24 h of incubation. I) Ceftazidime, J) Cefepime, K) Imipenem and L) Meropenem.
