# Supplementary material for: AiiM Lactonase Strongly Reduces Quorum Sensing Controlled Virulence Factors in Clinical Strains of Pseudomonas aeruginosa Isolated From Burned Patients
Source: Front Microbiol. 2019 Nov 14;10:2657. doi: 10.3389/fmicb.2019.02657 (PMC6868103; doi:10.3389/fmicb.2019.02657)
Supplement: Supplementary file 3 [file Data_Sheet_3.docx]

| **Homoserine lactone (HSL)** | **Retention time (min)** | |
| --- | --- | --- |
|  | **Hydrolyzed** | **Non-hydrolyzed** |
| **C4** | 1.42 | 2.29 |
| **C10** | 3.48 | 9.9 |
| **3OC8** | 1.11 | 1.86 |
| **3OC10** | 1.89 | 4.48 |
| **3OC12** | 2.24 | 5.3 |

Table S2. Retention time expressed in minutes of different HSL, without (non-hydrolyzed) and with (hydrolyzed) 5 µg/mL AiiM.
